# Ependymin‐related protein 1 levels are elevated in children with obesity and correlated with metabolic disorders

**DOI:** 10.1002/pdi3.25

**Published:** 2023-08-14

**Authors:** Chunfeng Mou, Shan Liu, Yetao Luo, Yu Xue, Jia Liu, Dapeng Chen, Xiaoqiang Li, Han Wang

**Affiliations:** ^1^ Department of Nuclear Medicine Children's Hospital of Chongqing Medical University National Clinical Research Center for Child Health and Disorders Ministry of Education Key Laboratory of Child Development and Disorders Chongqing Key Laboratory of Pediatrics Chongqing Key Laboratory of Child Health and Nutrition Chongqing China; ^2^ Department of Nosocomial Infection Control Second Affiliated Hospital Army Medical University Chongqing China; ^3^ Department of Clinical Laboratory Children's Hospital of Chongqing Medical University National Clinical Research Center for Child Health and Disorders Ministry of Education Key Laboratory of Child Development and Disorders Chongqing Key Laboratory of Pediatrics Chongqing Key Laboratory of Child Health and Nutrition Chongqing China

**Keywords:** children with obesity, Ependymin‐Related Protein 1, metabolic associated fatty liver disease, metabolic disorders

## Abstract

Ependymin‐related protein 1 (EPDR1) has been found in the secretory body of adipocytes where it plays a role in lipid binding, transportation, and catabolism. The aim of this study was to investigate serum EPDR1 levels in children with obesity and normal‐weight children and to compare the levels of EPDR1 between children with obesity, with and without metabolic‐associated fatty liver disease (MAFLD). Thirty‐four normal‐weight children and 49 children with obesity (15 with MAFLD) were included in the study. Circulating EPDR1, IL‐1β, and TNF‐α were measured using enzyme‐linked immunosorbent assays. Anthropometric and biochemical measurements related to obesity, blood lipids, and insulin resistance were performed on all participants. The serum EPDR1 levels of children with obesity were significantly higher than those of the control group. There was no difference in EPDR1 levels between the patients with and without MAFLD. Circulating EPDR1 was positively correlated with body mass index (BMI), BMI z‐score, insulin, glucose, homeostatic model assessment insulin resistance index (HOMA‐IR), triglycerides, white blood cells, and neutrophils. Binary logistic regression analysis showed a significant increase in the odds ratio of obesity with increasing EPDR1 levels. EPDR1 is strongly associated with obesity and may also be associated with metabolic disorders. This trial is registered with ChiCTR2300070951.

## Graphical abstract

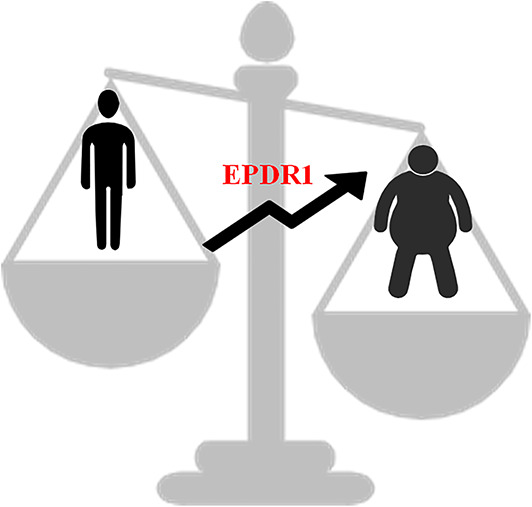

## 1 INTRODUCTION

Obesity is characterized by an abnormal or excessive accumulation of fat in adipose tissue and is influenced by genetic, lifestyle and environmental factors.^1^ It is a global issue and its prevalence in children is on the rise.^2^ Childhood obesity is a significant risk factor for endocrine disorders such as pubertas praecox, cardiovascular diseases, impaired glucose tolerance, insulin resistance, polycystic ovary syndrome, dyslipidemia, metabolic syndrome, and metabolic associated fatty liver disease (MAFLD).^3^, ^4^, ^5^, ^6^ Approximately 85% of school‐age children with obesity will remain obese into adulthood and are more likely to develop early‐onset complications such as hypertension, hyperglycemia, and hyperlipidemia. MAFLD is currently the most common cause of chronic liver disease in children in the United States and is closely linked to obesity.^7^, ^8^ It encompasses various liver diseases, including progressive steatosis, inflammation, fibrosis, cirrhosis, and eventually hepatocellular carcinoma.^9^ Therefore, identifying reliable biomarkers for obesity is crucial for early diagnosis and preventive strategies aimed at reducing the burden of adulthood.

Obese individuals typically exhibit chronic low‐grade inflammation, referred to as metabolic inflammation. Obesity increases the levels of lipopolysaccharide binding protein, which promotes the release of pro‐inflammatory factors, leading to elevated levels of inflammatory markers in the blood.^10^ Studies have shown that levels of several inflammatory markers in the blood of obese people are altered, including a significant increase in the levels of pro‐inflammatory factors such as Sub WISP1, interleukin‐6 (IL‐6), tumor necrosis factor (TNF‐α), leptin, and resistin as well as a significant decrease in levels of anti‐inflammatory factors such as adiponectin, omentin, and adipolin. These inflammatory factors regulate the interaction between adipose tissue and metabolic organs. For instance, MAFLD is often associated with abdominal obesity and may increase the risk of liver cirrhosis and liver cancer.^11^ Both MAFLD and obesity‐induced insulin resistance can increase the production of IL‐6 and TNF‐α.^12^

Ependymin‐related protein 1 (EPDR1) is a member of the ependymin family and is found in lysosomes and secretomes of most vertebrates. EPDR1 is a glycoprotein involved in several physiological and pathological processes, such as maintaining energy homeostasis, thermogenic differentiation, cell adhesion, cellular immunity, and inflammation.^13^, ^14^, ^15^ It contains hydrophobic binding grooves and is a LolA/EPDR folding protein. EPDR1 also acts as an activator protein or transporter of neuronal lipids, playing a crucial role in lipid binding, transportation, and catabolism. Recent research has shown that EPDR1 can interact with anionic, negatively charged liposomes such as bis(monoacylglycerol)phosphate (BMP) or ganglioside GM1 at an acidic Ph.^16^, ^17^ It stimulates neuraminidase‐3 activity and inhibits neuraminidase‐4. At the same time, some studies suggest that EPDR1 may play a role as a lysosome activating protein.^18^ In cultured cells, EPDR1 has been detected in the secretomes of fibroblasts and adipocytes.^14^, ^19^

MAFLD is strongly associated with obesity, but no previous studies have investigated whether EPDR1 is associated with obesity in children with or without MAFLD. The primary aim of this study was to investigate the levels of EPDR1 and the potential relationship between serum EPDR1 levels and metabolic parameters in both obese and normal‐weight children. The secondary objective was to compare EPDR1 levels between children with obesity with and without MAFLD.

## 2 MATERIALS AND METHODS

### 2.1 Subjects

In our study, we enrolled 83 children, of which 49 were classified as the obesity group (33 boys and 16 girls), while 34 age‐matched healthy children (18 boys and 16 girls) were included as the control group. The healthy children were selected from the Physical Examination Center of the Children's Hospital of Chongqing Medical University. The important standard guide on the health of preschool children issued by the National Health and Family Planning Commission "Screening for overweight and obesity among school‐age children and adolescents", which includes a table of BMI screening thresholds for overweight and obesity in 6–18 year old school‐age children and adolescents. BMI was calculated as weight (in kilograms) divided by the square of height (in meters). The exclusion criteria included metabolic syndrome such as hyperglycemia, hypertension, and dyslipidemia as well as any respiratory system, digestive system, or other diseases that could interfere with the research participation. The presence of MAFLD was often identified by abdominal ultrasonography that showed enhanced hepatic echo compatible with fatty infiltration of the liver. Various biomarkers such as alanine aminotransferase (ALT) and aspartate transaminase (AST) were also measured, with or without elevated levels. Children with obesity were categorized into two groups based on the liver brightness on ultrasonography: those with MAFLD (*n* = 15) and those without (*n* = 34).

### 2.2 Clinical and biochemical measurements

In our study, all subjects fasted for 10–12 h prior to participating in a series of body composition and anthropometry examinations. Two to 3 mL of venous blood were collected from each subject on an empty stomach and then centrifuged at 4°C with 3500 rpm for 10 min. The resulting serum was stored at −80°C for subsequent testing. Trained staff recorded age, height, and weight of all the subjects. Standard enzymatic assays were used to measure the levels of total cholesterol (TC), triglycerides (TG), low‐density lipoprotein cholesterol (LDL‐C), high‐density lipoprotein cholesterol (HDL‐C), gamma‐glutamyl transpeptidase (GGT), alanine aminotransferase (ALT), and aspartate aminotransferase (AST). Automated hematology analyzer (SYSMEX XE‐2100L, Japan) was used to measure white blood cells (WBC) and neutrophils. Fasting insulin (Fins) was measured using chemiluminescence (DiaSorin S.p.A. LIALSON, Italy). Fasting blood glucose (FBG) was detected using the glucose oxidase method. The formula for the homeostasis model assessment of insulin resistance (HOMA‐IR) is as follows: HOMA‐IR = FBG (mmol/L) × Fins (mU/L)/22.5.

### 2.3 Measurements of serum EPDR1, IL‐1β, and TNF‐α

Circulating EPDR1 concentrations were measured using a commercial ELISA kit (Catalog No. MBS9316185, MyBioSource, Inc, San Diego, USA), following the manufacturer's instructions. The detection range of the kit was 31.2–1000.0 ng/ml, with a minimum detectable dose of 5.0 ng/ml. Both intra‐assay CV (%) and inter‐assay CV (%) were less than 15%. Serum IL‐1β levels were measured using commercial ELISA kits (Catalog No. MM‐0181H1, Jiangsu Meimian Industrial Co., Ltd, Jiangsu, China). The detection range of this kit was 1.0–40.0 pg/ml with intra‐assay CV (%) and inter‐assay CV (%) less than 10% and 12%, respectively. Serum TNF‐α levels were measured using commercial ELISA kits (Catalog No. MM‐0122H1, Jiangsu Meimian Industrial Co., Ltd, Jiangsu, China). The detection range of this kit was 20.0–400.0 ng/L with intra‐assay CV (%) and inter‐assay CV (%) less than 10% and 12%, respectively.

### 2.4 Statistics

Continuous variables were presented as mean and standard deviation if they followed a normal distribution, or as median with interquartile range if they were non‐normally distributed. Differences between continuous variables were assessed using parametric tests (unpaired *t*‐test) if the distribution was normal, while nonparametric tests (Mann–Whitney *U* test) were used if the distribution was non‐normal. Categorical variables were expressed using Chi‐square tests or Fisher test. The Kolmogorov–Smirnov test was used to determine the distribution of the data. Multiple linear stepwise regression analysis was performed to identify variables that were independently correlated with serum EPDR1 levels. Spearman correlation analysis was used to examine the associations between serum EPDR1 levels and other variables. Predictive values of circulating EPDR1 for obesity were analyzed using receiver operating characteristic (ROC) curves. Binary logistic regression analysis was used to analyze the association of EPDR1 with obesity. Statistical analysis was performed using Social Sciences (SPSS) version 23.0. All results with a *p* value less than 0.05 were considered statistically significant.

## 3 RESULTS

### 3.1 The clinical and laboratory characteristics of all subjects

There were no significant differences in the sex distribution and we only included children aged between 6 and 9 years as study subjects. Table 1 reports the clinical and laboratory characteristics of the two groups. Children with obesity had significantly increased BMI, BMI z‐score, Fins, FBG, HOMA‐IR, TG, GGT, WBC, and neutrophils compared to the control group. However, there was no significant difference in serum levels of TC, LDL‐C, ALT, IL‐1β, and TNF‐α but higher HDL‐C and AST levels were observed in children without obesity (Table 1). Moreover, in children with obesity, those with MAFLD had higher BMI, BMI z‐score, Fins, HOMA‐IR, ALT, AST, and GGT but lower HDL‐C levels than those without MAFLD (Table 2).

**TABLE 1:** Comparison of clinical and laboratory parameters between control group and obese group.

Parameters	Control group (*n* = 34)	Obesity group (*n* = 49)	*p* Value
Gender (M/F)	18/16	33/16	0.185
Age (year)	7.00 (6.00–9.00)	8.00 (6.50–9.00)	0.268
BMI (kg/m^2^)	15.58 (14.83–16.86)	26.02 (22.32–27.83)	**<0.001**
BMI z‐score	−0.03 (−0.44–0.66)	3.13 (2.53–3.75)	**<0.001**
Fins (mU/L)	8.95 (5.15–11.43)	18.80 (13.40–32.95)	**<0.001**
FBG (mmol/L)	4.84 ± 0.40	5.14 ± 0.46	**0.002**
HOMA‐IR	1.74 (1.08–2.46)	4.22 (2.94–7.53)	**<0.001**
TG (mmol/L)	0.78 (0.69–0.83)	1.13 (0.82–1.42)	**<0.001**
TC (mmol/L)	4.06 ± 0.65	4.13 ± 0.96	0.711
HDL‐C (mmol/L)	1.44 (1.24–1.62)	1.23 (1.04–1.52)	**0.011**
LDL‐C (mmol/L)	2.40 ± 0.64	2.51 ± 0.83	0.518
ALT (U/L)	16.00 (14.00–20.00)	18.00 (14.00–25.50)	0.209
AST (U/L)	30.24 ± 6.58	25.10 ± 8.74	**0.005**
GGT (U/L)	6.00 (6.00–7.00)	15.00 (12.00–22.50)	**<0.001**
EPDR1 (μg/ml)	178.50 (134.25–212.25)	229.00 (189.50–325.00)	**<0.001**
IL‐1β (pg/L)	93.50 (70.00–287.75)	88.00 (72.00–362.50)	0.820
TNF‐α (pg/ml)	78.50 (63.75–190.50)	82.00 (66.00–219.50)	0.785
WBC (×10^9^/L)	6.29 (5.74–7.55)	8.24 (6.03–10.19)	**0.001**
Neutrophils (×10^9^/L)	2.96 (2.25–3.68)	4.50 (3.38–5.75)	**<0.001**

*Note*: Data are shown as means ± SD or median (interquartile range). Statistcal significance (*p* < 0.05) is indicated by bold values.

Abbreviations: ALT, alanine aminotransferase; AST, aspartate aminotransferase; BMI, body mass index; FBG, fasting blood glucose; Fins, fasting insulin; GGT, gamma‐glutamyl transpeptidase; HDL‐C, high‐density lipoprotein cholesterol; HOMA‐IR, the homeostasis model assessment of insulin resistance; IL‐1β, interleukin‐1β; LDL‐C, low‐density lipoprotein cholesterol; TC, total cholesterol; TG, triglyceride; TNF‐α, tumor necrosis factor‐α; WBC, white blood cells.

**TABLE 2:** Comparison of clinical and laboratory parameters between obese group with or without MAFLD.

Parameters	Without MAFLD group (*n* = 34)	With MAFLD group (*n* = 15)	*p* Value
Gender (M/F)	20/14	13/2	0.097
Age (year)	8.00 (6.00–9.00)	8.00 (7.00–9.00)	0.802
BMI (kg/m^2^)	24.57 ± 4.32	28.28 ± 2.64	**0.004**
BMI z‐score	2.95 (2.31–3.36)	3.43 (3.20–4.26)	**0.009**
Fins (mU/L)	16.30 (12.08–27.93)	27.80 (18.80–38.90)	**0.011**
FBG (mmol/L)	5.12 ± 0.44	5.20 ± 0.52	0.559
HOMA‐IR	3.67 (2.64–6.38)	5.61 (4.17–8.96)	**0.014**
TG (mmol/L)	1.13 ± 0.41	1.27 ± 0.64	0.338
TC (mmol/L)	4.16 ± 1.00	4.05 ± 0.90	0.719
HDL‐C (mmol/L)	1.29 (1.03–1.52)	1.12 (1.04–1.33)	0.340
LDL‐C (mmol/L)	2.40 (2.08–2.78)	2.45 (1.92–3.42)	0.610
ALT (U/L)	15.50 (13.00–19.25)	26.00 (23.00–43.00)	**<0.001**
AST (U/L)	23.00 (18.00–25.25)	27.00 (20.00–40.00)	**0.015**
GGT (U/L)	13.00 (12.00–16.25)	23.00 (21.00–28.00)	**<0.001**
EPDR1 (μg/ml)	226.50 (186.50–321.00)	253.00 (188.00–338.00)	0.618
IL‐1β (pg/L)	87.00 (69.00–187.75)	110.00 (78.00–1128.00)	0.233
TNF‐α (pg/ml)	81.00 (65.75–169.50)	96.00 (70.00–725.00)	0.380
WBC (×10^9^/L)	8.06 ± 2.50	8.91 ± 2.30	0.266
Neutrophils (×10^9^/L)	4.47 ± 1.54	5.01 ± 1.59	0.269

*Note*: Data are shown as means ± SD or median (interquartile range). Statistical significance (*p* < 0.05) is indicated by bold values.

Abbreviations: ALT, alanine aminotransferase; AST, aspartate aminotransferase; BMI, body mass index; FBG, fasting blood glucose; Fins, fasting insulin; GGT, gamma‐glutamyl transpeptidase; HDL‐C, high‐density lipoprotein cholesterol; HOMA‐IR, the homeostasis model assessment of insulin resistance; IL‐1β, interleukin‐1β; LDL‐C, low‐density lipoprotein cholesterol; TC, total cholesterol; TG, triglyceride; TNF‐α, tumor necrosis factor‐α; WBC, white blood cells.

### 3.2 Comparison of serum EPDR1 levels between different groups

**FIGURE 1:**
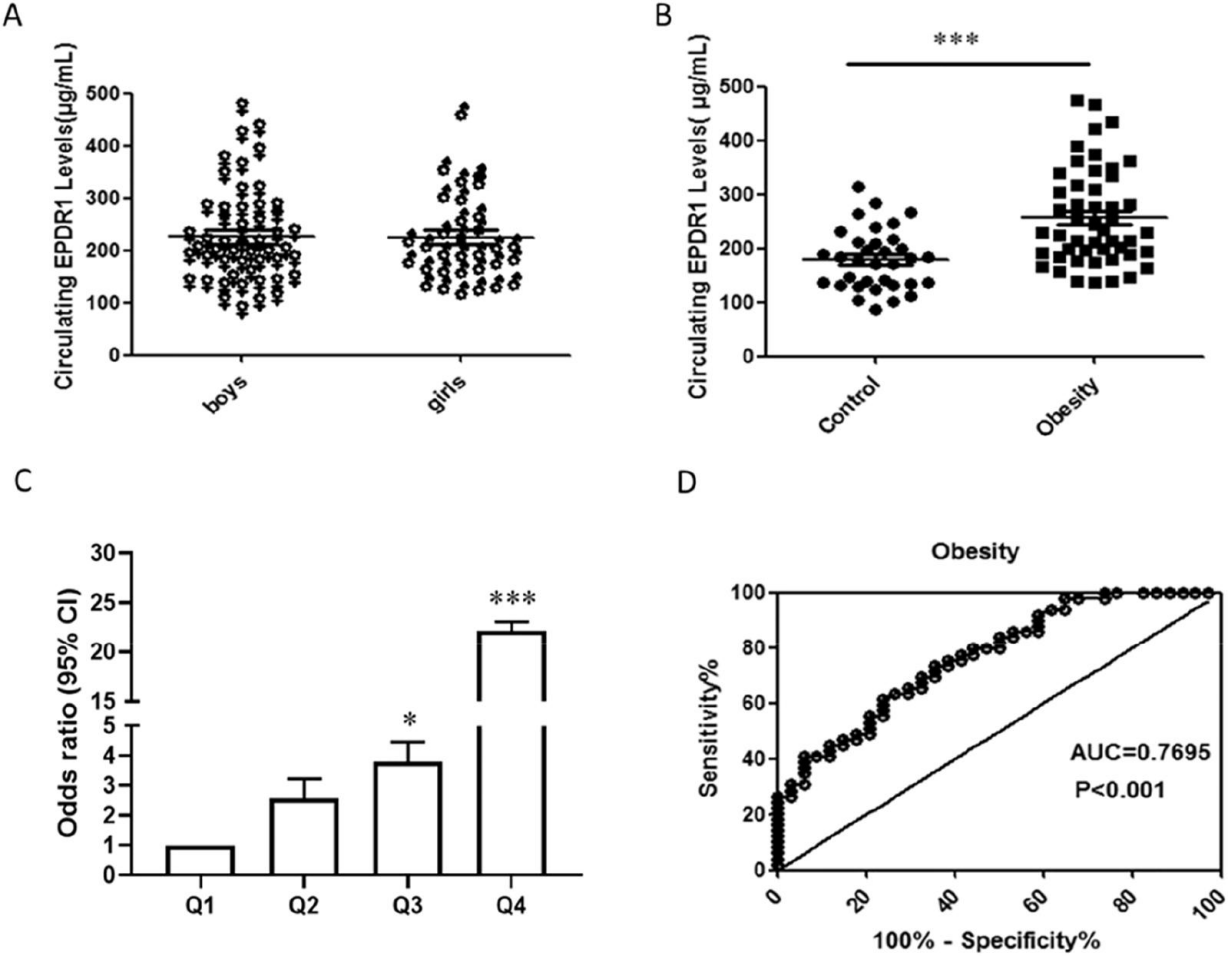
Circulating EPDR1 levels and ROC curve analysis in the study cohort. (A) Circulating EPDR1 levels were no difference between boys and girls (*p* = 0.955). (B) Circulating EPDR1 levels in children with obesity and normal‐weight controls (vs. control: ∗∗∗ *p* < 0.001). (C) Prevalence of elevated obesity in different quartiles of EPDR1: quartile 1, <166.0 μg/ml; quartile 2, 166.0–201.0 μg/ml; quartile 3, 201.0–276.0 μg/ml; quartile 4, >276.0 μg/ml (vs. quartile1: ∗ *p* < 0.05, ∗∗∗ *p* < 0.001). (D) ROC curve analyses for the prediction of obesity according to the EPDR1 levels.

In the whole cohort, the analysis of data showed that there was no significant difference in serum EPDR1 expression between boys and girls (201.00 (157.00–277.00) versus 205.50 (167.25–269.50)) μg/ml, *p* > 0.05 (Figure 1A). However, fasting serum EPDR1 levels were significantly higher in children with obesity than in normal‐weight children (229.00 (189.50–325.00) versus 178.5 (134.25–212.25)) μg/ml, *p* < 0.001 (Figure 1B). There was also no significant difference in serum EPDR1 levels between children with obesity with and without MAFLD (Table 2).

### 3.3 Associations between serum EPDR1 levels and metabolic parameters

We conducted a Spearman correlation analysis to investigate the relationship between serum EPDR1 concentration and other variables. The results showed that serum EPDR1 levels were positively correlated with BMI, BMI z‐score, Fins, FBG, HOMA‐IR, TG, white blood cells, and neutrophils, while negatively correlated with HDL‐C (Table 3). After adjusting for sex, serum EPDR1 level remained positively associated with BMI, BMI z‐score, Fins, FBG, HOMA‐IR, TG, white blood cells, and neutrophils and negatively associated with HDL‐C (Table 3). Multiple stepwise regression analysis revealed that BMI was the primary determinant of circulating EPDR1 with a beta‐coefficient of 0.384 (Table 3).

**TABLE 3:** Correlation analysis between EPDR1 and biochemical data in all participants.

Variables	EPDR1		EPDR1 (gender‐adjusted)		Multivariate	
	*r*	*P*	*r*	*P*	*b*	*P*
Age (year)	−0.025	0.823	−0.025	0.821	一	一
BMI (kg/m^2^)	0.384	**<0.001**	0.401	**<0.001**	0.384	**<0.001**
BMI z‐score	0.349	**0.001**	0.354	**0.001**	一	一
Fins (mU/L)	0.473	**<0.001**	0.475	**<0.001**	一	一
FBG (mmol/L)	0.301	**0.006**	0.307	**0.005**	一	一
HOMA‐IR	0.488	**<0.001**	0.491	**<0.001**	一	一
TG (mmol/L)	0.307	**0.005**	0.310	**0.005**	一	一
TC (mmol/L)	−0.149	0.178	−0.149	0.181	一	一
HDL‐C (mmol/L)	−0.224	**0.042**	−0.227	**0.040**	一	一
LDL‐C (mmol/L)	−0.174	0.116	−0.174	0.117	一	一
ALT (U/L)	−0.054	0.625	−0.057	0.610	一	一
AST (U/L)	−0.193	0.080	−0.195	0.079	一	一
GGT (U/L)	0.176	0.112	0.182	0.102	一	一
IL‐1β (pg/L)	0.116	0.297	0.116	0.299	一	一
TNF‐α (pg/ml)	0.106	0.338	0.106	0.341	一	一
WBC (×10^9^/L)	0.279	**0.011**	0.286	**0.009**	一	一
Neutrophils (×10^9^/L)	0.413	**<0.001**	0.424	**<0.001**	一	一

*Note*: In the multiple linear stepwise regression analysis, the values included for analysis were BMI, FBG, TG, HDL‐C, and WBC. Statistical significance (*p* < 0.05) is indicated by bold values.

To further investigate the relationship between EPDR1 and obesity, we divided serum EPDR1 levels into four quartiles based on its concentration in the entire cohort (Q1, <166.0 μg/ml; Q2, 166.0–201.0 μg/ml; Q3, 201.0–276.0 μg/ml, and Q4, >276.0 μg/ml). We then conducted binary logistic regression analysis to calculate the odds ratio of obesity. As expected, most individuals in the control group were in Q1 (41.2%), followed by 29.4% in Q2, 23.5% in Q3, and only a small proportion in Q4 (5.9%). However, the percentage of children with obesity was the highest in Q4 (38.8%), and the proportion in Q3 (26.5%) was higher than in Q2 (22.4%) and Q1 (12.3%) (Table 4). We found that the odds ratios for obesity significantly increased with increasing EPDR1 quartiles (Figure 1C). For example, the odds of having obesity in children with the highest level of EPDR1 (Q4) were 22 times higher than in those with the lowest level of EPDR1 (Q1) (Table 4).

**TABLE 4:** Distribution of patients according to EPDR1 quartile.

EPDR1 quartile	Control group	Obese group	Or (95% CI)	*p* Value
Q1	41.2%	12.3%	1	0.005
Q2	29.4%	22.4%	2.57 (0.71–9.27)	0.150
Q3	23.5%	26.5%	3.79 (1.03–13.91)	0.045
Q4	5.9%	38.8%	22.17 (3.88–126.65)	<0.001

*Note*: Data are shown as percentages of the total number in each group according to EPDR1 quartile. OR denotes odds ratio of obese children compared with the group of children with the lowest ranges of concentration of EPDR1; the likelihood of obesity increases as the level of serum concentration of EPDR1 increases.

Abbreviations: CI, confidence ratio; EPDR1, ependymin‐related protein 1; OR, odds ratio; Q, quartile.

### 3.4 Receiver operating characteristic analysis for obesity

To further clarify the relationship between EPDR1 and obesity, we conducted ROC curve analysis of serum EPDR1 levels to predict obesity. The area under the ROC curve was 0.770 (*p* < 0.001) with a sensitivity of 73.5% and specificity of 64.7% (Figure 1D). The best cut‐off value for EPDR1 to predict obesity was 193.5 μg/ml.

## 4 DISCUSSION

In this study, we investigated the fasting serum levels and biochemical characteristics of EPDR1 in normal weight and obese children. Our findings showed that serum EPDR1 levels were significantly elevated in children with obesity compared to those with normal weight. Previous research by Feng et al. identified EPDR1 as a protein related to adipogenesis^20^ and it was shown to interact with anionic negatively charged lipids and activate neuraminidase‐3 while inhibiting neuraminidase‐4,^18^ suggesting its potential function as a lipid transporter or lysosomal activator protein.^15^ Further research demonstrated that EPDR1 is a novel batokine that plays an important role in brown fat commitment. Several batokines have been reported to have a hormonal function, enhancing BAT activity, improving glucose metabolism, or mediating browning of white fat.^21^, ^22^, ^23^ Quantitative analysis also confirmed that EPDR1 secretion in brown adipocytes was higher than that in white adipocytes, suggesting that EPDR1 is selectively secreted by brown adipocytes but can also be produced by white adipocytes. However, EPDR1 knockout mice studies have revealed that EPDR1 affects metabolism without enhancing BAT activity.^14^

At the same time, we investigated the fasting serum EPDR1 levels and their biochemical characteristics in normal‐weight and obese children, as well as in obese children with and without MAFLD. Compared to normal‐weight children, we found that children with obesity had significantly higher serum EPDR1 levels. Previous research has suggested that EPDR1 may function as a lipid transporter or lysosomal activator protein and may be important for brown fat commitment. However, in our study, there was no significant difference in circulating EPDR1 levels between obese children with and without MAFLD. Instead, we found that liver function markers such as ALT, AST, and GGT were higher in obese children with MAFLD compared to those without. In the entire cohort, serum EPDR1 levels were positively correlated with BMI, which was higher in obese children with MAFLD compared to those without. EPDR1 was also shown to be downregulated during short‐ and long‐term weight loss.^24^ Additionally, obesity was usually associated with a low‐level chronic inflammatory state, marked by an increase in systemic inflammatory markers.^25^ We found that leukocyte and neutrophil counts were slightly higher in children with obesity but levels of IL‐1β and TNF‐α were not statistically significant. We speculate that the high levels of EPDR1 in patients with obesity may be related to BMI and inflammatory status but further studies with larger sample sizes are needed to confirm these preliminary findings.

To investigate the relationship between EPDR1 and obesity further, we analyzed the prevalence of obesity across different quartiles of circulating EPDR1 levels. Our findings showed that the relative risk of obesity increased significantly with increasing EPDR1 levels. The odds of obesity were 22 times higher in the group with the highest EPDR1 levels compared to the group with the lowest levels. Finally, we assessed the correlation between serum EPDR1 and obesity using ROC curves, which revealed that circulating EPDR1 could serve as a valuable marker for predicting obesity.

There are some limitations and shortcomings in our study. Firstly, the serum samples we collected were only from children aged 6–9 years in Southwest China and thus may not represent other populations. Secondly, the sample size was limited and outliers may have influenced our results. Lastly, our study is cross‐sectional and does not establish a causal relationship between increased circulating EPDR1 levels and the occurrence and development of obesity. To better understand the relationship between EPDR1 and obesity, further studies with larger sample sizes and longitudinal designs are needed. Nevertheless, our data provide clinical evidence suggesting that elevated serum EPDR1 may be linked to metabolic disorders and may have a potential role in the pathogenesis of obesity.

## 5 CONCLUSIONS

In summary, our data provides clinical evidence that elevated serum EPDR1 may play a potential role in the development of obesity and may be associated with metabolic disorders. Therefore, the concentration of serum EPDR1 may serve as a biomarker for predicting the risk of obesity.

## AUTHOR CONTRIBUTION

Chunfeng Mou, Han Wang conceived and designed the experiments, performed the experiments, analyzed the data, prepared figures and/or tables, authored or reviewed drafts of the paper, approved the final draft. Yetao Luo conducted statistical analysis of the data in the manuscript and approved the final draft. Shan Liu, Yu Xue, and Jia Liu performed the experiments, analyzed the data, prepared figures and/or tables, and approved the final draft. Dapeng Chen, Xiaoqiang Li authored or reviewed drafts of the paper, reviewed and edited the manuscript, and approved the final draft.

## CONFLICT OF INTEREST STATEMENT

The authors declare that there is no conflict of interest that could be perceived as prejudicing the impartiality of the research reported.

## ETHICS STATEMENT

This study was conducted according to the Declaration of Helsinki and approved by the Ethics Committee of Children's Hospital of the Chongqing Medical University (2022 ethical review No (495)). This trial is registered with ChiCTR2300070951.

## Data Availability

Data sharing not applicable to this article as no datasets were generated or analyzed during the current study.
